# Partial Shoulder Arthroplasty Guided by Three-dimensional Prototyping

**DOI:** 10.1055/s-0042-1749625

**Published:** 2022-06-29

**Authors:** Lucas Maia, Kennedy Tavares Ladeia, Bernardo Figueira Althoff, Adriano Marchetto, Diego Meneghel, Guilherme Valdir Baldo

**Affiliations:** 1 Divisão de Cirurgia de Ombro e Cotovelo, Pontifícia Universidade Católica de Campinas (PUC), Campinas, São Paulo, Brasil; 2 Divisão de Cirurgia de Mão, Pontifícia Universidade Católica de Campinas (PUC), Campinas, São Paulo, Brasil; 3 Divisão de Cirurgia de Ombro e Cotovelo, Instituto Wilson Mello, Campinas, São Paulo, Brasil; 4 Divisão de Cirurgia de Pé e Tornozelo, Escola Paulista de Medicina da Universidade Federal de São Paulo, São Paulo, Brasil; 5 Divisão de Cirurgia de Ombro e Cotovelo, Centro Universitário para o Desenvolvimento do Alto Vale do Itajaí (UNIDAVI), Rio do Sul, Santa Catarina, Brasil

**Keywords:** arthroplasty, replacement, shoulder, shoulder joint, three-dimensional imaging, three-dimensional printing

## Abstract

Three-dimensional (3D) printing technology is a reality in medicine. In Orthopedics and Traumatology, 3D printing guides a precise and tailored surgical treatment. Understanding and disseminating its applicability, use, and outcomes can foster academicism and improve patient care. This is a report of a rare case of a female young adult patient with osteonecrosis of the humeral head due to avascular necrosis developed in early childhood. The treatment was tailored and optimized with 3D printing, which helped determine the steps for partial humeral arthroplasty.

## Introduction

The emergence of three-dimensional (3D) printing technology is an industrial milestone. 1 Its system of adding layers by automation using some raw materials, based on pre-formulated prototypes, reinvented the means of production. 2 It spread to medicine quickly, with applications in various fields, including surgery, reconstruction, and rehabilitation, in addition to the manufacture of synthetic tissues, prostheses, implants, and anatomical models. 3 In orthopedics, 3D printing is used in a vast number of conditions, traumas, and sequelae with bone deformities. The use of prototypes designed from computed tomography or magnetic resonance imaging provides a new scenario for diagnosis and treatment in Orthopedics.

With 3D printing, it is possible to create anatomical models, facilitating the understanding of anatomical nuances and helping with surgical planning, including access routes, osteotomies, implant positioning, prostheses preparation, orthotics, and personalized implants. 4 Studies show that preoperative planning of personalized orthopedic implants using 3D prototypes improves the understanding of fracture pattern when compared to two-dimensional or 3D tests visualized on screen. 5 We present a case of avascular necrosis of the humeral head in a female patient with a history of hemolytic disease of the newborn who underwent surgical treatment with a partial shoulder arthroplasty planned using 3D printing.

## Case Report

This study was approved by the institutional ethics committee under the number CAAE-38891420.5.0000.5453.

The patient was a 23-year-old woman with a history of hemolytic disease of the newborn. Her complicated delivery required blood transfusion and hospitalization in an intensive care unit for 40 days. On admission, she presented osteomyelitis of probable hematogenous origin affecting the left femur and humeral head, which was clinically managed.

**Fig. 1:**
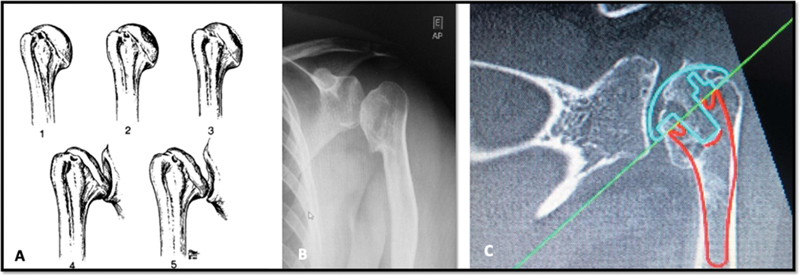
Humeral head osteonecrosis: (A) progressive Creuss 6 stages 1) pre-radiographic stage, 2) subchondral sclerosis, 3) subchondral fracture, 4) evident head collapse, 5) degenerative changes of the head; (B) preoperative radiography; (C) computed tomography scan.

During osteoarticular development, the patient presented changes at the left humeral head and femur. It is difficult to determine whether the changes were due to the infection, secondary complications during birth, or both. At 15-years-old, she underwent a left hip arthroplasty due to pain and loss of range of motion (ROM). At 23 years old, she had a 4 cm shortening of the left upper limb, in addition to pain and functional limitation during gait. On the left shoulder, she had adequate muscle trophism, and the following ROM values: 50° for abduction (ABD), 20° for internal rotation (IR), and 0° for external rotation (ER); her Disabilities of the Arm, Shoulder and Hand (DASH) score was 93 of 150, the American Shoulder and Elbow Surgeons (ASES) score was 10 for pain and 18 for function, and pain was rated as 9 to 10 using the visual analog scale (VAS). Radiographs showed humeral head flattening Creuss stage 4 6 and an intact glenoid, with a medullary canal size of approximately 1.4 cm ( Figs. 1A and 1B ).

A computed tomography scan was used for surgical planning and demonstrated the need for a humeral prosthesis with a fracture nail due to its smaller diameter compared to conventional nails ( Fig. 1C ).

**Fig. 2:**
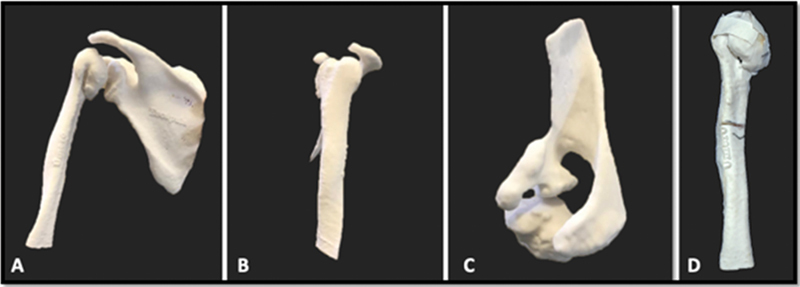
Full-size, 3D-printed prototype: (A) rear aspect; (B) lateral aspect; (C) top aspect; (D) anteromedial aspect of the humerus (post-test).

**Fig. 3:**
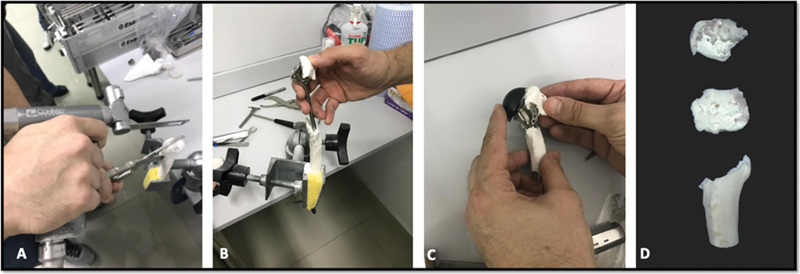
(A) Osteotomy of the humeral head and tuberosities; (B) insertion of the intramedullary nail; (C) verification of the greater tuberosity positioning; (D) post-test outcome at the proximal end of the humerus.

Despite the scan, we chose a more precise planning method due to the patient's age. The Renato Archer Information Technology Center (CTI) prepared a 3D-printed prototype using selective laser sintering printing, polyamide PA 12, and a 0.1 mm resolution level between layers. Reconstruction was performed with the Invesalius and Magics (CTI, Campinas, SP, Brazil) software ( Fig. 2 ). The piece was taken to the laboratory for surgical planning and an Equinoxe Fracture System (Exactech, Inc., Gainesville, FL, USA) prosthesis was chosen. The need for a greater tuberosity (GT) osteotomy was detected during the procedure as an initially unplanned surgical step ( Fig. 3 ).

**Fig. 4:**
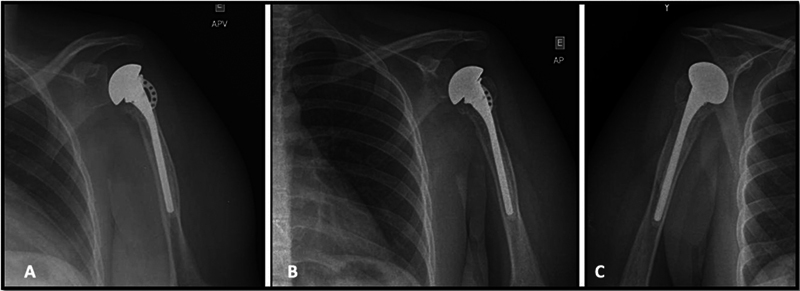
Immediate postoperative radiograph: (A) true anteroposterior view; (B) anteroposterior view; (C) oblique view.

Surgery was performed using a deltopectoral approach, with greater and lesser tuberosity osteotomy. The canal was prepared using a humeral reamer, followed by manual insertion of the orthopedic cement, and application of a 6.5 mm x 120 mm fracture nail. A 1.5 mm offset replicator plate and a 38 mm x 16 mm torque with short humeral head were inserted and sutured to the tuberosities using Fiberwire 2.0 (Arthrex Inc., Naples, FL, USA). The surgery took 1 hour and 23 minutes, and the bleeding was minimal. The patient used a sling for 3 weeks and then physical therapy started. Fig. 4 shows radiographic outcomes.

**Fig. 5:**
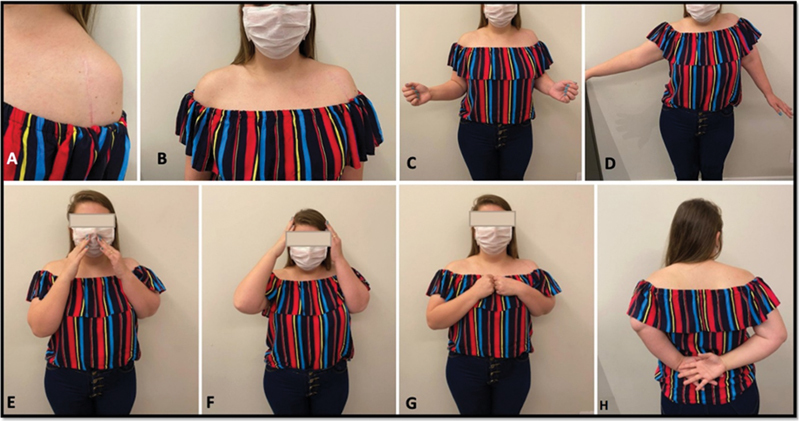
Postoperative clinical outcomes: (A) surgical incision; (B) frontal appearance; (C) external rotation; (D) abduction; (E) hand to mouth; (F) hand to head; (G) internal rotation; (H) hand to back.

At a follow-up visit, 1 year and 3 months after surgery, the patient presented good trophism and functional shoulder mobility. The following values were detected on examination: 60° ABD, 20° ER, and 30° IR, as shown in Fig. 5 . Scores improved for the DASH to 30 of 150, ASES to 0 (pain) and 26 (function), and VAS to 0.

## Discussion

Glenohumeral conditions, such as avascular osteonecrosis in young patients, require greater therapeutic engagement due to the higher life expectancy and demand. Conservative treatment is the first line. Arthroscopic debridement can delay joint survival. Joint replacement surgeries are indicated when symptoms affect daily activities. Although total arthroplasties have been gaining space, hemiarthroplasties in young patients with a functional glenoid cavity remain a good indication. 6 7

Orfaly et al. 8 presented a series of 21 shoulders with a diagnosis of avascular necrosis of the humeral head submitted to partial arthroplasty with significant improvement in pain (average VAS from 88 to 16 in the whole series, *p* < 0.01), function by the average ASES from 36 to 88 ( *p* < 0.001), and ABD went from 88 to 123. Smith et al. 9 evaluated 32 partial arthroplasties for corticosteroid-associated osteonecrosis with 13 excellent outcomes (42%), four satisfactory outcomes (13%), and 14 unsatisfactory outcomes (45%).

According to the current literature, planning with 3D printing improves shoulder surgery by clarifying the following parameters: compliance, retroversion and glenoid diameter, bone stock, joint surface thickness, posterior offset, and medial offset. 10 11 12 At a recent literature review, Cordona et al. demonstrated that: (1) while planning total and reverse arthroplasties, the glenoid component is the one benefiting the most from the technique, both in terms of implant and bone stock anticipation; (2) in instabilities, printing can design implants to cover specific bone defects; (3) in trauma, it helps the detailed recognition of the fracture pattern, facilitating the surgical procedure. The authors conclude that the technique is beneficial and can reduce surgical time, blood loss, and fluoroscopy use. 13

The purpose of planning with a 3D-printed prototype in a young female patient with distorted anatomy was to perform all the steps quickly, accurately, and safely, and to increase prosthesis survival. However, an unforeseen extra step, a GT osteotomy, was required, potentially leading to implant choice issues and iatrogenic injuries, such as periprosthetic fractures and increased safety for the surgery team during the procedure. Such cases highlight the benefit of clarifying anatomical particularities, reducing surgical time and its implications. Although ABD did not show significant improvement (in addition to difficulties in carrying out rehabilitation due to the COVID-19 pandemic), quality of life and functional scores improved.

Three-dimensional printing for orthopedic planning is a reality. Treatment customization, a better understanding of anatomy, and reduced surgical time are well-established benefits, and its use must be considered for cases in which the anatomy raises questions in planning. The increased use and spread of 3D printing will improve its availability as a surgical tool.
