# Supplementary material for: Partial Shoulder Arthroplasty Guided by Three-dimensional Prototyping
Source: Rev Bras Ortop (Sao Paulo). 2022 Jun 29;59(Suppl 1):e73–7. doi: 10.1055/s-0042-1749625 (PMC11254443; doi:10.1055/s-0042-1749625)
Supplement: Related texts

# Research article: Artroplastia parcial de ombro guiada por prototipagem tridimensional

**Keywords:** articulação do ombro, artroplastia do ombro, imageamento tridimensional, impressão tridimensional

## Abstract

A tecnologia de impressão tridimensional (3D) é uma realidade no âmbito da medicina. Na Ortopedia e Traumatologia, a impressão 3D direciona um tratamento cirúrgico preciso e individualizado. Compreender e difundir suas aplicações, utilidades e resultados pode fomentar o academicismo e melhorar o cuidado ao paciente. Trata-se de relato de um caso raro de uma paciente adulta jovem com osteonecrose da cabeça umeral por necrose avascular desenvolvida na primeira infância. O tratamento foi individualizado e otimizado com a impressão 3D, que ajudou a determinar os passos para o procedimento de artroplastia parcial de úmero.

## Introdução

O surgimento da tecnologia da impressão tridimensional (3D) é considerado um marco industrial. 1 Seu sistema de adição de camadas por automação, com determinadas matérias primas, a partir de protótipos pré-formulados reinventou a forma de produção. 2 Rapidamente se difundiu na medicina, com aplicações em diversos campos como: cirúrgico, reconstrução e reabilitação, a partir da fabricação de tecidos sintéticos, próteses, implantes e modelos anatômicos. 3 Na ortopedia, vem ao encontro da vasta quantidade de doenças, traumas e sequelas que apresentam deformidades ósseas. O uso de protótipos projetados a partir de imagens por tomografia computadorizada ou ressonância magnética proporciona um novo cenário para o diagnóstico e tratamento dentro da especialidade.

Com a impressão 3D, torna-se possível criar modelos anatômicos, facilitando a compreensão das nuances anatômicas, auxiliando com o planejamento cirúrgico, incluindo vias de acesso, osteotomias, posicionamento de implantes, elaboração de próteses, órteses e implantes personalizados. 4 Estudos apontam que o planejamento pré-operatório para cirurgias ortopédicas a partir de moldes 3D trazem uma maior compreensão do padrão da fratura quando comparado com exames em 2D ou 3D visualizados em tela. 5 Apresentamos um caso de necrose avascular da cabeça de úmero em uma paciente com histórico de doença hemolítica do recém-nascido, que foi submetida ao tratamento cirúrgico com artroplastia parcial de ombro com planejamento usando impressão 3D.

## Relato de Caso

Este estudo foi aprovado pelo comitê de ética da instituição sob o número CAAE-38891420.5.0000.5453.

Paciente, 23 anos, feminino, histórico de doença hemolítica do recém-nascido. Parto complicado com necessidade de transfusão sanguínea e internação em unidade intensiva por 40 dias. Na internação apresentou osteomielite de provável origem hematogênica, acometendo a cabeça femoral e umeral esquerda, manejado clinicamente.

Durante o desenvolvimento osteoarticular observaram-se alterações na cabeça do úmero e do fêmur esquerdo. É difícil inferir se as alterações foram devido ao quadro infeccioso, complicações secundárias durante o nascimento ou ambos. Aos 15 anos, foi submetida a artroplastia do quadril esquerdo decorrente à dor e perda de amplitude de movimento (ADM). Aos 23 anos apresentava 4cm de encurtamento do membro superior esquerdo, com dor e limitação funcional durante a marcha. No ombro esquerdo, apresentava adequado trofismo muscular, e os seguintes valores de ADM: abdução (ABD) de 50°, rotação interna (RI) de 20° e rotação externa (RI) de 0°. O resultado do questionário de Disabilities of the Arm, Shoulder and Hand (DASH) foi de 93 em 150, do questionário American Shoulder and Elbow Surgeons (ASES) foi de dor 10 e função 18 e a escala visual da dor (EVA) marcou de 9 a 10. Radiografia apresentou achatamento da cabeça umeral Creuss 4 6 e glenóide preservada, com um canal medular de aproximadamente 1,4cm ( Figs. 1A e 1B ).

**Fig. 1:** Osteonecrose da cabeça de úmero: (A) estágios progressivos de Creuss 6 1) estágio pré-radiográfico, 2) esclerose subcondral, 3) fratura subcondral, 4) colapso evidente da cabeça, 5) alterações degenerativas da cabeça; (B) radiografia pré-operatória; (C) navegação com tomografia computadorizada.

Utilizou-se navegação tomográfica para planejamento cirúrgico, demonstrando necessidade de prótese umeral com haste de fratura, devido a diâmetro menor que as hastes convencionais ( Fig. 1C ).

Apesar da navegação, optou-se por um planejamento mais preciso, por conta da idade da paciente. Realizada a prototipagem Impressão 3D pelo Centro de Tecnologia da Informação (CTI) Renato Archer com impressão sinterização seletiva a laser, material poliamida PA 12 e resolução entre as camadas de 0,1 mm. A reconstrução foi feita pelo software Invesalius e Magics (CTI, Campinas, SP, Brasil) ( Fig. 2 ). A peça foi levada para laboratório e realizado o planejamento cirúrgico e a prótese escolhida foi uma Equinoxe Fracture System (Exactech, Inc., Gainesville, FL, EUA). Durante o procedimento, percebeu-se a necessidade de osteotomia da grande tuberosidade (GT) passo cirúrgico inicialmente não planejado ( Fig. 3 ).

**Fig. 2:** Protótipo 3D impresso em tamanho normal: (A) vista posterior; (B) vista lateral; (C) vista superior; (D) visão ânteromedial do úmero (pós-teste).

**Fig. 3:** (A) Osteotomia da cabeça umeral e tuberosidades; (B) inserção da haste intramedular; (C) verificação do posicionamento da grande tuberosidade; (D) resultado pós teste da extremidade proximal do úmero.

A cirurgia foi realizada utilizando acesso deltopeitoral, com osteotomia do tubérculo maior e menor. O canal foi preparado através da fresa umeral, seguida pela inserção manual do cimento ortopédico e aplicação da haste de fratura 6,5 × 120 mm. Foram inseridas a placa replicadora offset 1,5 mm e o parafuso de torque e cabeça umeral curta 38 × 16 mm e sutura dos tubérculos através de fios Fiberwire 2.0 (Arthrex Inc., Naples, FL, EUA). Tempo cirúrgico foi de 1 hora e 23 minutos, com sangramento mínimo. A paciente manteve a tipóia por três semanas e depois iniciou a fisioterapia. Resultados radiográficos conforme a Fig. 4 .

**Fig. 4:** Radiografia pós-operatória imediata: (A) posição anteroposterior verdadeira; (B) posição anteroposterior; (C) oblíqua.

Em um seguimento de 1 ano e 3 meses apresentava bom trofismo e mobilidade funcional do ombro. Os seguintes resultados foram observados no exame: ABD de 60°, RE de 20° e RI de 30° conforme as imagens clínicas ( Fig. 5 ) Apresentou melhora do DASH de 30 em 150, ASES para dor 0 e função 26, e EVA para 0.

**Fig. 5:** Resultados clínicos pós-operatórios: (A) incisão cirúrgica; (B) aparência frontal; (C) rotação externa; (D) abdução; (E) mão à boca; (F) mão à cabeça; (G) rotação interna; (H) mão às costas.

## Discussão

Condições glenoumerais, como osteonecrose avascular em pacientes jovens, exigem maior engajamento terapêutico devido à alta expectativa de vida e demanda. O tratamento conservador é a primeira linha. Desbridamentos artroscópicos podem aumentar a sobrevida articular. Quando os sintomas prejudicam as atividades diárias, cirurgias de substituição articular são indicadas. Embora artroplastias totais venham ganhando espaço, as hemiartroplastias em pacientes jovens com a cavidade glenoidal funcional ainda permanecem uma boa indicação. 6 7

Orfaly et al. 8 apresentaram uma série de 21 ombros com diagnóstico de necrose avascular da cabeça umeral, submetidos a artroplastia parcial com melhora importante das dores (EVA médio de 88 para 16 em toda a série, *p* < 0,01), da função pelo ASES médio de 36 para 88 (p < 0,001) e ABD foi de 88 para 123. Smith et al. 9 avaliaram 32 artroplastias parciais por osteonecrose associada a corticóide com 13 resultados excelentes (42%), 4 satisfatórios (13%) e 14 insatisfatórios (45%).

De acordo com a literatura atual, a cirurgia do ombro se favorece do planejamento com impressão 3D ao permitir uma maior nitidez dos seguintes parâmetros: conformidade, retroversão e diâmetro glenoidal, estoque ósseo, espessura da superfície articular, offset posterior, offset medial. 10 11 12 Em uma recente revisão de literatura sobre o tema, Cordona et al. demonstraram: (1) no planejamento de artroplastias totais e reversas, o componente glenoidal é que mais se beneficia da técnica tanto quanto a antecipação do implante quanto ao estoque ósseo; (2) nas instabilidades onde a impressão pode projetar implantes para cobrir defeitos ósseos específicos; (3) no trauma, em que auxilia o reconhecimento detalhado do padrão da fratura, facilitando o ato cirúrgico. Concluem que a técnica é benéfica e responsável pela redução do tempo cirúrgico, da perda sanguínea e de fluoroscopia demasiada. 13

O propósito do planejamento da impressão 3D em um caso com a anatomia distorcida em uma paciente jovem foi executar de maneira rápida, precisa e segura todos os passos, e proporcionar uma maior sobrevida à prótese. Entretanto, demonstrou-se a necessidade de um passo extra não previsto, uma osteotomia da GT, que potencialmente causaria imprevistos na escolha de implante, lesões iatrogénicas como fraturas periprotéticas e permitiu maior segurança da equipe de cirurgia durante o procedimento. Casos como este salientam os benefícios decorrentes do esclarecimento das particularidades anatômicas, redução de tempo cirúrgico e suas implicações. Apesar da ABD não ter apresentado melhora expressiva (além de dificuldades na realização de reabilitação devida à pandemia COVID-19), houve melhora da qualidade de vida e dos escores funcionais.

A impressão 3D para o planejamento ortopédico é uma realidade. Personalização do tratamento, melhor compreensão da anatomia e redução do tempo cirúrgico são benefícios bem estabelecidos, e seu uso deve ser considerado em casos nos quais a anatomia gera dúvidas durante o planejamento terapêutico. Quanto mais utilizada e difundida, mais disponível estará como arsenal cirúrgico.
